# Nitinol Staples for Olecranon Osteotomy Fixation, Juxtacortical Versus Inset, Effect on Biomechanical Stability

**DOI:** 10.1016/j.jhsg.2021.03.004

**Published:** 2021-04-27

**Authors:** Ajay Mahenthiran, Ethan Kacena-Merrell, Weinong W. Chen, Boon Him Lim, Hannah Dineen

**Affiliations:** ∗ Indiana Hand to Shoulder Center, Indianapolis, IN

**Keywords:** Olecranon, Osteotomy, Troughing, Staples

## Abstract

**Purpose:**

Hardware prominence is a concern in the fixation of olecranon osteotomies. Staple fixation has provided low-profile secure fixation in other areas of orthopedics. Without insetting, staples still have subcutaneous prominence. This study examines whether nitinol staples, when inset into bone via cortical notching, in an olecranon osteotomy can provide fixation strength sufficient for daily activities.

**Methods:**

Olecranon osteotomies were created in 8 cadaver arms and fixed with 2 nitinol staples. For inset and juxtacortical (noninset) staples, a micrometer measured the displacement between preplaced proximal and distal wires for 3 increasing loads: 0 N, 15 N, and 150 N. This measurement reflected the loss of osteotomy compression. We placed each arm in a pneumatic machine that flexed the elbow from 0° to 90° for 500 cycles at each load. We performed a 2-tailed *t* test (α value 0.05, β value 0.2) to evaluate for differences in the loss of compression between inset and noninset nitinol staples.

**Results:**

We performed the displacement measurement procedure for both staple types at each of the 3 loads. At 0 N, the average displacement of inset was 0 mm and that of noninset was 0.02 mm. At 15 N, the average displacement of inset was 0.02 mm and that of noninset was 0.04 mm. At 150 N, the average displacement of inset was 0.05 mm and that of noninset was 0.09 mm. When comparing the displacement at the 3 force loads, there were no statistically significant differences between the staple types (*P* = .323).

**Conclusions:**

This study shows that inset staples do not considerably weaken osteotomy fixation with nitinol staples. Thus, nitinol staples may provide a low-profile, operatively-efficient fixation method compared with tension-band or screw-and-plate fixation methods for olecranon osteotomies. Future research can include comparing staples with plate constructs.

*Type of study/level of evidence*: Therapeutic III.

The optimal fixation method for recovery following an olecranon osteotomy remains controversial. Regardless of the method of fixation (plates or tension band), hardware prominence and soft tissue irritation have been problematic for patients following an olecranon osteotomy. As much as 30% of olecranon osteotomy hardware has been removed as it irritated the patients.^1^^,2^ Using nitinol staples to secure an olecranon osteotomy could improve this issue by providing strong fixation with a low-profile design. In addition, nitinol staples have already been used in a variety of applications for bone stabilization, including calcaneocuboid fusions, talonavicular fusions, and osteosynthesis of the scaphoid.^3^^,^^4^ Schnabel et al^5^ have demonstrated that nitinol compression staples have greater biomechanical stability than the tension-band wiring technique in transverse patellar fractures.

Furthermore, this study tests for a difference between insetting the staples and placing them juxtacortical to the olecranon. To minimize subcutaneous prominence, cortical troughing to allow staples to be inset flush to the bone may minimize the soft tissue irritation and patient discomfort on the posterior elbow. However, there is a concern that cortical notching for insetting the staples may weaken the fixation. A previous study of cortical notching used a sawbone model without cyclic loading to assess the effect of notching on mechanical stability.^6^ One of these studies demonstrated that troughing of the bone does not significantly diminish the biomechanical properties of the construct.^6^ This could potentially help improve the problem of subcutaneous prominence associated with hardware that creates increased patient discomfort following olecranon osteotomies. Thus, we hypothesized that there would be no notable decrease in biomechanical stability when the staples are troughed and inset into the bone instead of being placed on the top of the cortex. Our primary objective was to measure osteotomy displacement under progressive loads.

## Methods

**Figure 1:**
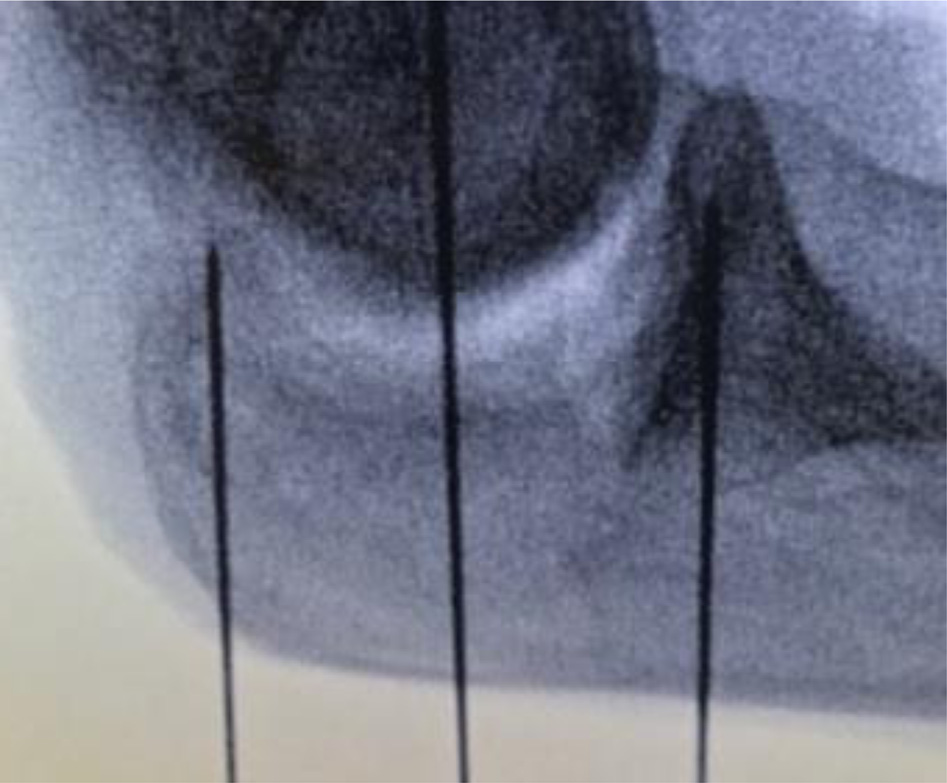
Fluoroscopy scan of the positioning of 2 K-wires placed at the frontal and distal ends of the olecranon. The third wire identifies the midpoint of the osteotomy.

We used 8 fresh-frozen cadaver arms (Appendix 1, available on the *Journal’s* website at www.jhsgo.org). To prepare the cadaver arms, we cut down to the bone and removed surrounding soft tissue while leaving tendons and ligaments intact for experimentation. We placed two 1.6-mm (0.063-in) K-wires in the cadaver arm, with 1 placed into the proximal olecranon aiming toward the tip and 1 placed distally in the ulna aiming toward the coronoid tip, ensuring that the K-wires were inserted orthogonal to the axis of the olecranon. We used the midpoint between the 2 wires as the apex of the chevron cut to standardize the size and position of the osteotomy (Fig. 1).

Using a surgical bone saw with a blade thickness of 0.51 mm, we made an angled chevron cut in the olecranon with the apex set at the midpoint between the 2 K-wires placed at the proximal and distal ends of the olecranon. We placed a longitudinal K-wire through the olecranon to secure the reduction during staple placement (BME Synthes), and then the wire was removed for testing. Using the staple drill guide, we drilled 2 holes for the staple legs that were proximally and distally equidistant from the osteotomy. For the inset group of staples, we used a rongeur with jaws equivalent in width to the staple. The trough was progressively deepened until it equaled the staple bridge’s 2-mm depth, allowing for the flush placement of the staple. This positioning allowed for a notch to be removed from the cortex between the 2 drill holes with the exact width and depth necessary for complete insetting of the staple flush with the bone surface. Using the preloaded insertion device, the staples (Depuy Synthes BME Elite) were inserted into the predrilled holes and then released from the insertion device. After the release from the inserter, they were gently tapped into place to be countersunk in the trough. The staples started in a state of stress and changed to a state of compression after the insertion. The inserter was designed to prestress the staple, and on release, it applied continuous compression.

For the staples that were noninset, we did not notch the cortex. We placed 1 staple in the medial third and 1 in the lateral third of the olecranon. We measured the distance between the proximal and distal K-wires with a micrometer after staple placement but before applying the progressive series of fixed forces to the elbow.

To compare the effect of troughing most directly, we chose the same thickness and brand of staples for both inset and noninset techniques. Since a trough (3-mm deep and 5.5-mm wide) was removed from the cortex between the staple drill holes, we wanted the staples’ effective length in the cancellous bone to be consistent. In the test with noninset staples, we used an 18-mm leg-length staple, but only 15 mm of the leg was in cancellous bone, leaving 3 mm in the cortex. Therefore, we used staples with 15-mm leg length for the staples that were inset.

**Figure 2:**
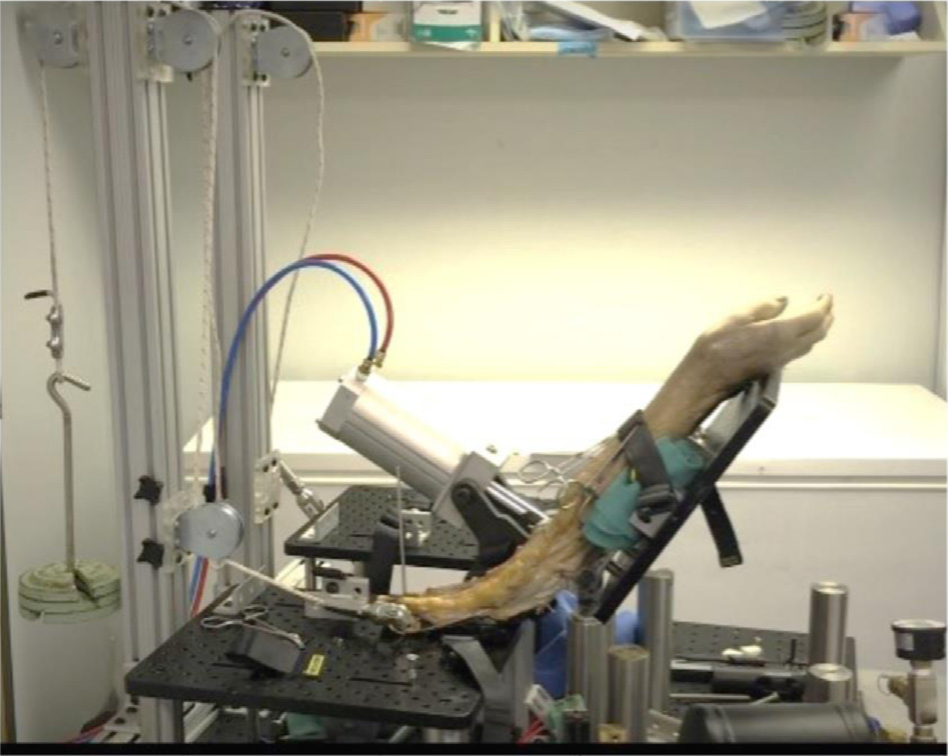
A custom arm-cycling machine developed by the Impact Science Laboratory in the School of Aeronautics and Astronautics at Purdue University. We used this machine for the range of motion activities that stimulated the olecranon.

The cadaver arms were then placed in a custom arm-cycling machine with a progressive series of fixed weights attached to the triceps through a braided suture (Fig. 2). A total of 8 cadaver arms were used: 4 for the inset group and 4 for the noninset group of staples. This number was a sample size of convenience, and we performed the 4 noninset constructs consecutively, followed by the 4 inset staple constructs.

The pneumatic machine flexed the elbow from 0° to 90°. A range of motion from 0° to 90° reflected an elbow’s active range of motion that some postoperative protocols included to minimize the terminal stress on the construct while helping minimize elbow stiffness. Therefore, we designed our testing apparatus to follow that limitation. The specimens were tested at 0 N, 15 N, and 150 N, with each load running for 500 cycles. After each set of 500 cycles, we used a micrometer to measure the distance between the 2 K-wires to assess any displacement. Measurements were taken without a load, with the arm flexed at 90°. Fluoroscopic images were also taken to visually assess any osteotomy offset. The maximum load to mimic force on the elbow during active range of motion was 150 N.^7^

We performed a 2-tailed *t* test (α value 0.05, β value 0.2) to evaluate for differences in the loss of compression between inset and noninset nitinol staples. The 2-tailed test was used as we theoretically could not identify in advance which construct would be more robust.

## Results

At 0 N, the average displacement of inset was 0 mm and that of noninset was 0.02 mm. At 15 N, the average displacement of inset was 0.02 mm and that of noninset was 0.04 mm. At 150 N, the average displacement of inset was 0.05 mm and that of noninset was 0.09 mm. Table 1 and Table 2 provide tabularized versions of all data. When comparing the displacement between inset and noninset staples, there were no statistical differences at any force level.

**Table 1:** Displacement of Noninset Nitinol Staples

Specimen	Total Displacement of 2 Noninset Nitinol Staples After 500 Runs at 0 N ± 0.01 mm	Total Displacement of 2 Noninset Nitinol Staples After 500 Runs at 15 N ± 0.01 mm	Total Displacement of 2 Noninset Nitinol Staples After 500 Runs at 150 N ± 0.01 mm
Specimen 1	0.00 mm	0.01 mm	0.02 mm
Specimen 2	0.00 mm	0.04 mm	0.04 mm
Specimen 3	0.03 mm	0.04 mm	0.10 mm
Specimen 4	0.04 mm	0.06 mm	0.18 mm
Averages	0.02 ± 0.02 mm	0.04 ± 0.02 mm	0.09 ± 0.06 mm

**Table 2:** Displacement of Inset Nitinol Staples

Specimen	Total Displacement of 2 Inset Nitinol Staples After 500 Runs at 0 N ± 0.01 mm	Total Displacement of 2 Inset Nitinol Staples After 500 Runs at 15 N ± 0.01 mm	Total Displacement of 2 Inset Nitinol Staples After 500 Runs at 150 N ± 0.01 mm
Specimen 1	0.00 mm	0.02 mm	0.06 mm
Specimen 2	0.00 mm	0.02 mm	0.04 mm
Specimen 3	0.00 mm	0.02 mm	0.06 mm
Specimen 4	0.01 mm	0.02 mm	0.02 mm
Averages	0.00	0.02 ± 0.00 mm	0.05 ± 0.02 mm

## Discussion

The fixation of olecranon osteotomies has been a controversial topic in the field of orthopedics. The problem is that current olecranon fixation methods have resulted in considerable patient discomfort after surgery. With the current olecranon fixation methods, as much as 30% of olecranon osteotomy hardware has been removed as it irritated the patients.^2^ Thus, finding a less prominent construct able to withstand the forces of early active motion could be beneficial. Insetting staples flush with the cortical surface would likely cause less hardware irritation than a noninset staple, thus providing greater comfort to the patients in the postoperative period.

Through experimentation, we found that insetting the staples and minimizing hardware prominence was as biomechanically robust as its noninset counterpart. Compared with staples placed on the cortex, notching of the cortex did not demonstrate a statistically significant difference (*P* value = .323) in osteotomy movement with a progressive series of fixed repetitive loads. There was also no difference noted fluoroscopically.

According to McKnight et al,^6^ troughing of the bone did not significantly diminish a construct’s biomechanical properties in a sawbones model. Here, we identified similar findings in a cadaver model. With cyclic progressively-increasing loads, we found no statistically significant (*P* value = .323) compromise of fixation from notching the cortex to allow for flusher staple inset. We did not design our study to show the amount of olecranon osteotomy motion that could be tolerated while still achieving bony union. However, with a load of 150 N, the average movement at the osteotomy site of the troughed staples compared favorably with other fixation constructs.^7^ The load of 150 N was selected because it replicated the force applied to the triceps for active range of motion.

Furthermore, a previous study on symptomatic hardware removal after olecranon fixation revealed an incidence of 29% in the olecranon locking plate-and-screws fixation cohort and 29.2% in the tension-band wire fixation cohort.^2^ Thus, symptomatic hardware prominence has been a problem in the past with previous olecranon osteotomy fixation techniques. In demonstrating the biomechanical stability of nitinol staples, our results suggest that inset staples can provide reasonable fixation strength while having less subcutaneous prominence when used for osteotomy fixation. A further area of potential investigation would be a single staple construct. If it were biomechanically sufficient, it would provide even greater intraoperative efficiency and lower cost compared with the 2-staple construct that we used. Another potential exploration on this topic would be using varying leg lengths and bridge widths to accommodate the practicality of working with different bone sizes during surgical operations.

When comparing the staple fixation strength with the tension-band fixation strength, Hammond et al^7^ tested elbows with tension-band fixation at 150 N after 500 cycles and found ranges of posterior displacement from 0.5 mm to 1.8 mm. In contrast, our average displacement for 150 N testing after 500 cycles for the inset staples was 0.05 mm and that for the noninset staples was 0.09 mm.^7^ However, there are not any currently available studies for the comparison of data to the plate fixation technique with a similar testing mechanism, which is a limitation of this study.

This study has other limitations as well. Namely, the elbows range within flexion and extension planes only from 0° to 90°. We believe this is the most common range of motion employed by patients within the immediate postoperative period. Increased forces beyond 90° could result in increased displacement not identified in this study. The sample size also limits the results. Using more cadavers could help confirm the validity of the results. Heavier loads may produce a different result, but we do not feel that most of the patients in the early postoperative period are allowed to subject their arms to heavier loads. Although a micrometer measurement may be a limitation of the study, it is accurate to 1/100th of mm, likely less than the clinically meaningful difference.

The investigation has shown that staples inset into the bone via cortical notching do not notably weaken osteotomy fixation. Nitinol staples may provide a low-profile, operatively-efficient fixation method compared with the traditional tension-band fixation method for olecranon osteotomy. Future research on this topic can be performed to compare the use of plate constructs to the double-staple construct used in this investigation.
