# Supplementary material for: Nitinol Staples for Olecranon Osteotomy Fixation, Juxtacortical Versus Inset, Effect on Biomechanical Stability
Source: J Hand Surg Glob Online. 2021 Apr 27;3(4):172–5. doi: 10.1016/j.jhsg.2021.03.004 (PMC8991424; doi:10.1016/j.jhsg.2021.03.004)
Supplement: Appendix 1 [file mmc1.docx]

**Appendix:**

|  | Weight (in lbs) | Age | Gender |
| --- | --- | --- | --- |
| Specimen 1 (inset) | 198 | 84 | Male |
| Specimen 2 (inset) | 226 | 73 | Female |
| Specimen 3 (inset) | 325 | 63 | Male |
| Specimen 4 (inset) | 185 | 67 | Male |
| Specimen 1 (noninset) | 225 | 74 | Male |
| Specimen 2 (noninset) | 174 | 69 | Female |
| Specimen 3 (noninset) | 240 | 67 | Male |
| Specimen 4 (noninset) | 198 | 84 | Male |
